# Genetic analysis of an allergic rhinitis cohort reveals an intercellular epistasis between FAM134B and CD39

**DOI:** 10.1186/1471-2350-15-73

**Published:** 2014-06-27

**Authors:** Rossella Melchiotti, Kia Joo Puan, Anand Kumar Andiappan, Tuang Yeow Poh, Mireille Starke, Li Zhuang, Kerstin Petsch, Tuck Siong Lai, Fook Tim Chew, Anis Larbi, De Yun Wang, Michael Poidinger, Olaf Rotzschke

**Affiliations:** 1 SIgN (Singapore Immunology Network), A*STAR (Agency for Science, Technology and Research), Singapore 138648, Singapore; 2 Doctoral School in Translational and Molecular Medicine (DIMET), University of Milano-Bicocca, Milan 20126, Italy; 3 Department of Biological Sciences, National University of Singapore, Singapore 117543, Singapore; 4 Department of Otolaryngology, National University of Singapore, Singapore 119228, Singapore

**Keywords:** Epistasis, Treg, Monocyte, eQTLs, Allergic rhinitis

## Abstract

**Background:**

Extracellular ATP is a pro-inflammatory molecule released by damaged cells. Regulatory T cells (Treg) can suppress inflammation by hydrolysing this molecule via ectonucleoside triphosphate diphosphohydrolase 1 (ENTPD1), also termed as CD39. Multiple studies have reported differences in CD39^+^ Treg percentages in diseases such as multiple sclerosis, Hepatitis B and HIV-1. In addition, CD39 polymorphisms have been implicated in immune-phenotypes such as susceptibility to inflammatory bowel disease and AIDS progression. However none of the studies published so far has linked disease-associated variants with differences in CD39 Treg surface expression. This study aims at identifying variants affecting CD39 expression on Treg and at evaluating their association with allergic rhinitis, a disease characterized by a strong Treg involvement.

**Methods:**

Cohorts consisting of individuals of different ethnicities were employed to identify any association of CD39 variants to surface expression. Significant variant(s) were tested for disease association in a published GWAS cohort by one-locus and two-locus genetic analyses based on logistic models. Further functional characterization was performed using existing microarray data and quantitative RT-PCR on sorted cells.

**Results:**

Our study shows that rs7071836, a promoter SNP in the CD39 gene region, affects the cell surface expression on Treg cells but not on other CD39+ leukocyte subsets. Epistasis analysis revealed that, in conjunction with a SNP upstream of the FAM134B gene (rs257174), it increased the risk of allergic rhinitis (*P* = 1.98 × 10^-6^). As a promoter SNP, rs257174 controlled the expression of the gene in monocytes but, notably, not in Treg cells. Whole blood transcriptome data of three large cohorts indicated an inverse relation in the expression of the two proteins. While this observation was in line with the epistasis data, it also implied that a functional link must exist. Exposure of monocytes to extracellular ATP resulted in an up-regulation of FAM134B gene expression, suggesting that extracellular ATP released from damaged cells represents the connection for the biological interaction of CD39 on Treg cells with FAM134B on monocytes.

**Conclusions:**

The interplay between promoter SNPs of CD39 and FAM134B results in an intercellular epistasis which influences the risk of a complex inflammatory disease.

## Background

Allergic Rhinitis (AR) is a common airway disease where allergen exposure triggers an IgE-mediated immune response. The typical symptoms include nasal itchiness, rhinorrhea, sneezing and progressive blockage of the inflamed nasal passages [1]. The disease is driven by a complex interplay of various leukocytes, including mast cells, eosinophils and basophils but also CD4+ T cells, IgE-producing B cells and dendritic cells. Th2 cytokines such as IL-4, IL-5 and IL-13 drive IgE production, promote eosinophil infiltration to the nasal mucosa, and stimulate mast cell release of key vasoactive mediators such as histamine [2-4]. In this context also monocytes are important effectors and regulators of inflammation [5]. While pro-inflammatory monocytes can fuel the allergic reaction by releasing cytokines such as TNF-α and IL-6, they can be converted into anti-inflammatory monocytes to dampen the reaction [6].

Central to the prevention or attenuation of pro-inflammatory immune responses are CD4+ Foxp3+ T regulatory cells (Treg). They can inhibit the proliferation of CD4+ effector T cells and impair the production of various Th2 cytokines [7-10]. A key mechanism by which Treg exert their regulatory function is the expression of the ectoenzyme CD39 [11,12]. CD39 is involved in the hydrolysis of extracellular ATP, which is typically released from cells following tissue damage. ATP-sensors such as P2X- and P2Y-receptors are important mediators of allergic airway inflammation and their blockade has been shown to strongly suppress allergic reactions in experimental asthma models [13,14]. Treg-expressed CD39 thus contributes to the control of inflammation via the removal of ATP [11,12]. Genetic and phenotypic correlations of CD39 variants have already revealed strong associations with inflammatory bowel disease [15] and multiple sclerosis [11,16], as well as with viral infections including HIV [17] and Hepatitis B [18]. However, a link between CD39 and allergic diseases has not previously been identified.

In the current report we demonstrate that variation in cell surface expression in Treg cells is associated with a genetic polymorphism (rs7071836) located in the promoter region of CD39. On its own this polymorphism had no direct impact on risk of AR but associated with disease risk through an epistatic interaction with rs257174, a promoter SNP of the *cis*-Golgi protein FAM134B. As rs257174 alters the gene expression in monocytes but not in Treg cells this represents the first example of an intercellular epistasis.

## Methods

### Ethics statement

This study has been approved by the Institutional Review Board of the National University of Singapore (IRB ref. NUS 07–023, NUS 10–343 and NUS 09–256) and complies with the Helsinki declaration. Written informed consent was obtained from all donors prior to sample collection.

### Study populations

#### ***Case–control cohorts***

We used two age-matched cohorts of Singapore Chinese individuals [19], including 456 AR cases and 486 non-atopic controls for investigative discovery and a separate cohort of 676 AR cases, 511 non-atopic controls and 1647 atopic individuals without AR symptoms for validating our statistical interaction and estimating the role played by atopy in the epistasis. These two cohorts were used to evaluate the role played by the variant rs7071836 in AR risk both in the context of disease association and epistatic phenomena. Details of sample collection for each cohort and the genotyping and quality control filters applied for the discovery cohort, are described in [19]. Cases were defined as individuals displaying a positive skin prick test for at least one of the two house dust mite allergens tested (*Dermatophagoides pteronyssinus, Blomia tropicalis*) and exhibiting two or more symptoms of nasal blockage, sneezing, nasal itching, and rhinorrhea. Controls were defined as skin-prick test negative individuals with no history of allergic disease or AR symptoms. All individuals were genotyped using the Illumina HumanHap 550 k BeadChip version 3 (Illumina, San Diego, California). Due to constraints in the number of available Sequenom slots 20 SNPs were selected for replication. Genotyping of the 20 SNPs chosen for replication in the validation cohort was performed using Sequenom’s MassARRAY system and iPLEX technology (Sequenom Inc, San Diego) on 2834 samples. The experiment was carried out according to the manufacturer’s guidelines. SNPs were called using the Sequenom TYPER software and were checked for deviation from Hardy-Weinberg equilibrium (variants with an adjusted P-value <0.05 in controls only or across all samples were excluded from the analysis; rs2900474, *P*_unaffected_ = 6.01 × 10^-10^; rs4862396, *P*_unaffected_ = 8.53 × 10^-4^).

#### ***Functional cohorts***

An independent cohort of 165 ethnic Chinese volunteers was recruited to validate the genotype-phenotype association between rs7071836 and CD39 protein expression. Samples were collected in a similar manner and were age- and gender-matched to the case–control cohorts. Genotyping was performed on a genome-wide scale using the Illumina HumanOmni5-Quad chip (Illumina, San Diego, California) on DNA extracted from blood following standard protocols. SNP calling was carried out using the Genome Studio genotyping module (Illumina, San Diego, California). The same quality control filters were applied as those described for the case–control cohorts.

A small cohort of 41 self-reported Chinese individuals and 22 self-reported Caucasian subjects was recruited internally and then phenotyped for CD39 protein expression by T regulatory cells to evaluate the relative frequency of the ‘CD39lo’ phenotype in each ethnic group.

#### ***Published whole blood cohorts***

Three published cohorts (Kora F4 [20], DILGOM [21], SHIP-TREND [22]) for which whole blood gene expression measurements were available were used to correlate CD39/FAM134B expression levels. The three cohorts are composed respectively of 993, 518 and 991 healthy individuals of Caucasian ethnicity. A detailed description of how each cohort was collected and samples were processed can be found in the respective publications. Gene expression processed values were downloaded from the corresponding public online repositories Array Express (E-MTAB-1708, E-TABM-1036) and Gene Expression Omnibus (GEO) (GSE36382).

### FACS analysis

In both functional cohorts, CD39 expression was determined by FACS staining of PBMCs pre-incubated with LIVE/DEAD Fixable Blue Dead Cell Stain kit (Invitrogen) to identify viable cells. The cells were then incubated with anti-CD39 APC (clone TÜ66), anti-CTLA-4 PE (clone BNI3), anti-CCR6 PerCP-Cy5.5 (clone 11A9), anti-CD4 APC-Cy7 (clone RPA-T4), anti-CD25 PE-Cy7 (clone M-A251), anti-CD45RA eFluor605 (clone H100) mAbs. Intracellular staining of Treg was conducted using the anti-FoxP3 eFluor450 (clone PCH101) Staining kit (eBioscience). Adult peripheral blood T cells either express CD45RA or CD45RO and few cells are double positive or double negative [23]. In our cohort, CD45RA- FoxP3+ CD25+ CD4+ T cells are termed as CD45RA- T regulatory (Treg) cells (CD45RO+) whereas CD45RA- FoxP3- CD25- CD4+ are CD4+ effector T (Teff) cells (CD45RO+). The level of CD39 expression was measured using a BD LSR II flow cytometer (BD Biosciences). The gating strategy for Treg, T effector cells, B cells and monocytes is outlined in Additional file 1: Figure S1. The variability of CD39 expression among human Treg from different donors was established by calculating the ratio of CD39 geometric mean fluorescence intensity relative to the CD39 geometric mean fluorescence of donor-matched B cells (which constitutively express high levels of CD39). Samples were classified as CD39 high expressing Treg (‘CD39hi’) or CD39 low expressing Treg (‘CD39lo’) using the unsupervised clustering method *k*-means on the log_2_-transformed ratios. The analysis was performed using the function *k*-means in R 2.15.1 [24], with the number of clusters set to two. Each population was considered and classified separately. CD39 expression values for the two groups as clustered by the *k*-means method are depicted in Additional file 2: Figure S2.

### Cell sorting

Human blood was collected into BD K_2_ EDTA vacutainers (Becton, Dickinson and Company) and the PBMCs were isolated by centrifugation over Ficoll-Paque density gradients (GE Healthcare) for 30 min at 400 × g. PBMCs were then re-suspended in FACS buffer (0.5% bovine serum albumin, 2 mM EDTA in PBS) and incubated at 4°C for 15 min with anti-CD49d FITC (clone MZ18-24A9, Miltenyi Biotec), CD127 PE (clone HIL-7R-M21), CCR6 PerCP-Cy5.5 (clone 11A9), CD4 APC-Cy7 (clone RPA-T4) CD25 PE-CY7 (clone M-A251), and CD19 Alexa700 (clone HIB19). All mAbs were purchased from BD Biosciences unless otherwise stated. After incubation, the cells were washed and re-suspended in FACS buffer at 1–1.5 × 10^7^ cells/ml for cell sorting of Treg, T effector cells, B cells and monocytes using a BD FACS Aria II cell sorter (BD Biosciences). See Additional file 3: Figure S3 for cell sorting strategy.

### Expression analysis

FACS-sorted Treg, T effector cells, B cells and monocytes were obtained from 15 healthy donor blood samples selected from the discovery cohort. Target ssDNA was prepared starting with 50 ng total RNA (RIN ≥ 7.1) using the Ambion WT Expression Kit and the Affymetrix WT Terminal Labelling kit. Fragmented ssDNA was hybridized to the Affymetrix Human Exon 1.0ST Arrays. The GeneChip arrays were washed and stained using the GeneChip Fluidics Station 450. After staining, the GeneChip arrays were scanned using a GeneChip Scanner 3000 at the BSF Microarray Facility. Array QC was conducted using the Affymetrix Expression Console Software. Raw data were normalized using the Robust Multi-Array Average (RMA) at the gene level [25].

### Monocyte ATP treatment

Monocytes from individuals heterozygous for rs257174 were purified from PBMCs by positive selection using MACS human CD14 MicroBeads (Miltenyi Biotec) according to manufacturer’s instructions. Monocytes were plated at 0.5 × 10^6^ cells/well in 24-well tissue culture plates and then incubated with or without cell culture-grade ATP disodium salt hydrate (Sigma-Aldrich) in RPMI-1640 supplemented with 10% fetal bovine serum, 2 mM L-glutamine, 1 mM sodium pyruvate, 100 units/ml of penicillin and 100 μg/ml of streptomycin. For the inhibition of ATP the incubation was carried out in the presence of 10 μM of the purinergic receptor inhibitor A-438079 hydrochloride (Tocris Bioscience). Monocytes were incubated for 2 h before harvesting using cell scrapers. The treated monocytes were stored in TRIzol (Invitrogen Life Technologies) at -80°C until further analysis.

### Genotyping

Genotyping was performed by PCR using SsoFast EvaGreen Supermix (Bio-Rad) on a CFX96 Real-Time System (Bio-Rad). DNA was isolated using DNeasy Blood & Tissue Kits (Qiagen) according to the manufacturer's instructions. The following primers were used for the analysis: FAM134B rs257174 forward primer: GCACGCTTTTGCCTTTGTAAT; FAM134B rs257174 reverse primer: CACCCACTGGGAGAAAAGAC. Amplification was carried out using the following protocol: 3 min at 95°C, 40 cycles of 5 s at 95°C, 5 s at 58°C, and final extension for 10 s at 95°C. A melt curve was generated from 65 to 95°C (in 0.2°C increments) with 10 s/step. Genotype analysis was performed using Bio-Rad Precision Melt Analysis software.

### Quantitative RT-PCR

Total RNA was isolated using TRIzol and RNeasy RNA isolation Kits (Qiagen) according to the manufacturer's instructions. Reverse transcription was performed using QuantiTect Reverse Transcription Kits (Qiagen). Expression analysis was performed by real-time PCR on a CFX96 Real-Time System (Bio-Rad). The analysis was carried out using the following protocol: 30 s at 95°C, 40 cycles of 5 s at 95°C, and ending with 5 s at 62°C. HPRT was used as the housekeeping reference gene for normalization. qRT-PCR was run using the following primers: FAM134B forward primer for the long isoform (Exon1,2): CTGCTGTTCTGGTTCCTTGC; FAM134B reverse primer for the long isoform (Exon1,2): CGCCCAAGTATCATGACGGA; FAM134B forward primer for the short isoform (Exon4,5): GCAGCCTTTGCCACTGTTATTAT; FAM134B reverse primer for the short isoform (Exon4,5): ATAACTTCCCAGCTTTTGCCTG; HPRT forward primer: CTCAACTTTAACTGGAAAGAATGTC; HPRT reverse primer: TCCTTTTCACCAGCAAGCT.

### Statistical analysis

Association analysis was performed using the command --logistic in PLINK v.1.07 [26] software including sex as a covariate (--sex). Due to the homogeneity of the cohort (which consisted of Singapore Chinese university students only), age and population were not included as factors in the model. Gene-gene interactions were evaluated in PLINK v.1.07 [26] conditioning on rs7071836 using the command --condition, fitting a logistic model (--logistic --interaction) and including sex as a covariate (--sex).

We fitted the following model:

$$Y\sim \beta_{0}+\beta_{1}A+\beta_{2}B+\beta_{3}\mathrm{AB}+\beta_{4}S+\epsilon$$

where A represents the allele dosage for the first SNP, B represents the allele dosage for the second SNP, AB is the interaction term, and S is the sex covariate.

Association between rs7071836 and CD39 cell surface expression was evaluated using Kruskal-Wallis testing on geometric mean intensity values. Association between genotypes and gene expression was evaluated using one-way ANOVA on log_2_-transformed expression values considering each genotype class as a distinct group (Graphpad Prism 6).

Differences in gene expression between control samples and samples treated with ATP were evaluated using repeated-measures ANOVA on ∆c(t) values with the assumption of sphericity. The mean of each group was compared to the mean of the control samples using Tukey's multiple comparisons test. Concentration specific effects were evaluated using the post test for linear trends in Graphpad Prism 6. Normality was assessed using the Shapiro-Wilk normality test as implemented by the function shapiro.test in R 2.15.1 [24]. Homoscedasticity was tested using the Bartlett test of homogeneity of variances as implemented by the function bartlett.test in R 2.15.1 [24]. Clustering of whole blood CD39 gene expression into three groups (CD39lo, CD39int, CD39hi) was performed using the unsupervised clustering method *k*-means as implemented by the function kmeans in R 2.15.1 [24] with the number of clusters set to three. Differences in FAM134B expression across the three groups (CD39lo, CD39int, CD39hi) were estimated using Kruskal-Wallis. Pairwise differences between CD39lo and CD39hi groups were tested using Dunn’s multiple comparison test (Graphpad Prism 6). Correlation coefficients and significance were computed using Spearman correlation and the fitting line was evaluated using linear regression (Graphpad Prism 6). Each cohort was analyzed independently.

Linkage disequilibrium plots were built using the software ArchiLD [27] on LD values estimated from the 1000 Genomes Pilot Project for CHB + JPT [28].

### Power estimation

Power estimation for epistatic interactions was performed using QUANTO software [29] with a significance threshold of 5 × 10^-8^ for the discovery study and 0.0025 for the validation study (Bonferroni correction for 20 tests). For both genes, the mode of inheritance was hypothesized to be log-addictive. Prevalence was set to 13% in both the discovery and validation cohorts. For each epistatic pair the allele frequency for the first SNP participating in the interaction was set to 0.25 (i.e. the allele frequency of rs7071836 in the discovery cohort) and the allele frequency of the second SNP was set to the allele frequency, in the discovery cohort, of the epistatic partner under consideration. R_GH_, the interaction effect, was set to the OR of the interaction in the discovery cohort.

## Results

### CD39 expression on Treg is influenced by promoter SNP rs7071836

**Figure 1:**
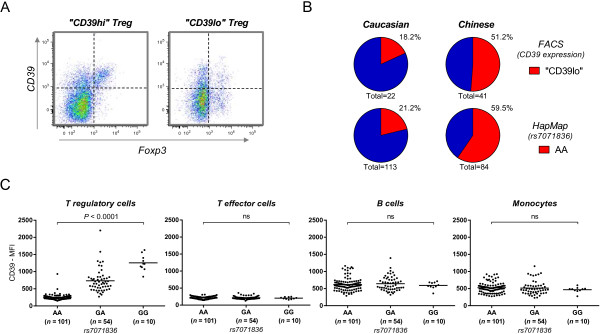
**Promoter SNP rs7071836 influences Treg expression of CD39. (A)** Illustrative example of the ‘CD39hi’ and ‘CD39lo’ Treg phenotypes. Intracellular staining of the Treg-associated transcription factor FoxP3 vs. cell surface expression of CD39 is shown for the CD45RO + CD4+ T cell compartment in two separate individuals of Chinese ethnicity. **(B)** Frequency of the ‘CD39lo’ Treg phenotype correlated with the frequency of the rs7071836-AA genotype (calculated using samples from HapMap3 release #27). **(C)** Genotype/phenotype association in a cohort of Chinese ethnicity (*n* = 165) showing CD39 expression in subsets of peripheral blood Treg, CD4+ effector T cells, B cells and monocytes. Significance was evaluated by Kruskal-Wallis test. Gating of the different cell populations is shown in Additional file 1: Figure S1.

In humans, CD39 is expressed by effector/memory-like Treg cells [11]. For this study we assessed CD39 cell surface expression in a small cohort of 41 Chinese and 22 Caucasian blood donors. As previously reported in Caucasians [11] we detected substantial inter-individual differences in CD39 surface expression in blood samples from volunteers of Chinese ethnicity (Figure 1A). However, while only ~20% of Caucasians were ‘CD39lo’, more than half of Chinese donors exhibited this phenotype (Figure 1B, upper panel). An analysis of CD39 SNP frequencies as reported for both ethnicities by the HapMap project [30-33] suggested that rs7071836 could be associated with the CD39 Treg phenotype (Figure 1B, lower panel). The frequency of the ‘CD39lo’ Treg phenotype in the Caucasian and Chinese cohorts closely resembled the frequency of the ‘AA’ genotype for this SNP.

Accordingly, FACS analysis of a larger cohort of 165 ethnic Chinese blood donors confirmed that rs7071836 SNP variants are indeed strongly associated with the phenotype of Treg cells (Figure 1C). Notably, the correlation was restricted to Treg, since CD39 expression on monocytes, B cells and T effector cells appeared unaffected by the allelic state of the SNP. In line with our finding a variant in strong linkage disequilibrium (LD) with rs7071836 was recently reported as being associated with the percentage of CD39+ activated CD4+ Treg in a Caucasian population [34].

### SNP rs7071836 tags a cluster of perfectly-linked SNPs

**Figure 2:**
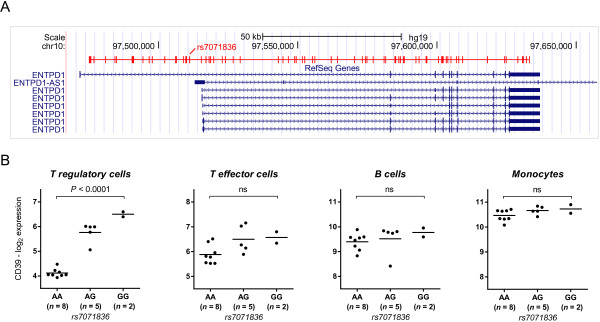
**Association of rs7071836 with CD39 mRNA levels in Treg but not other CD39+ human leukocytes. (A)** The position of the SNP-cluster which is in perfect linkage disequilibrium (r^2^ = 1) with rs7071836 in Asian individuals (CHB/JPT) is shown here with respect to the transcripts of the gene CD39 (ENTPD1). Linkage disequilibrium was estimated using the 60 samples sequenced by the 1000 Genomes Pilot Project. This SNP-cluster contains 82 SNPs spanning the entire gene. The position of the tag-SNP rs7071836 is indicated. **(B)** Impact of rs7071836 on CD39 gene expression in Treg, CD4+ T effector cells, B cells and monocytes isolated by FACS sorting from 15 genotyped individuals. The CD39 mRNA content is plotted with reference to rs7071836 genotype. Statistical analysis revealed that the polymorphism is associated with CD39 cell surface expression in T regulatory cells only (*P* < 0.0001). Gene expression was measured using the Affymetrix Human Exon 1.0ST Array and data were normalized using the Robust Multichip Average (RMA) at the gene level. P-values for the association were evaluated using one-way ANOVA on log_2_-transformed data (*n* = 15).

In Asian individuals rs7071836 tags a group of 82 perfectly-linked SNPs (SNP-cluster [27]) that cover the entire gene locus (Figure 2A). Some of these SNPs have been associated with risk of inflammatory disorders and viral infections. Specifically, the variants rs10748643 and rs11188513 have been respectively linked in Caucasians with risk of inflammatory bowel disease [15] and HIV [17]. In this population, both rs10748643 and rs7071836 are part of the same cluster, with linkage of r^2^ = 0.68 to rs11188513. mRNA analysis in FACS-sorted cells confirmed that the pattern of CD39 cell surface expression was directly reflected by the amount of mRNA detected in the respective cell types (Figure 2B). While CD39 mRNA levels in Treg cells varied according to rs7071836 genotype, expression levels in B cells, monocytes and CD4+ T effector cells were unaffected by the polymorphism.

### SNP rs7071836 affects AR risk via epistatic interaction with rs257174

To evaluate the role of the rs7071836 cluster in determining AR risk we assessed a Singapore Chinese cohort that comprised 456 atopic individuals affected by AR and 486 non-atopic asymptomatic controls (described in a previous publication) [19]. Using standard logistic regression models, we were unable to find any evidence of a direct association of rs7071836 with the risk of AR (*P* = 0.82, Table 1). We therefore explored possible epistatic interactions of rs7071836 with other polymorphic sites. Of the 550,000 SNPs detected by the Illumina HumanHap 550 k array, a total of 447,081 tag SNPs passed quality control assessment as described in [19]. Using a cut-off of 10^-4^ we identified 58 candidate interactions with rs7071836 that exhibited odds ratios (ORs) of 0.17 - 2.94 with P-values ranging from 10^-4^ to 10^-6^ (Additional file 4: Table S1).

**Table 1:** Association results for rs7071836 and AR in a Singapore Chinese population

**Gene**	**SNP**	**Test**	**Frequency cases**	**Frequency controls**	**Alleles**^**a**^	**MAF**^**b**^	**OR**^**c **^**[CI**^**d **^**95%]**	**P-value**
CD39	rs7071836	LOGISTIC	0.25	0.25	G/A	0.25	1.03 [0.83-1.27]	0.82

^a^Minor allele listed first. ^b^Minor Allele Frequency. ^C^Odds Ratio. ^d^Confidence Interval.

To identify the ‘true’ epistatic partners of rs7071836 an independent Singapore Chinese replication cohort consisting of 676 atopic AR cases and 511 non-atopic asymptomatic controls was employed [19]. Twenty SNPs were selected for replication and genotyped by Sequenom (Sequenom Inc, San Diego). After Bonferroni correction, the power to detect a significant interaction was estimated to be 0.88 - 0.99 (Additional file 5: Table S2). This finally allowed us to confirm an interaction of rs7071836 with a second SNP located ~12 kbp upstream of gene FAM134B (rs257174).

The combined P-value of discovery and validation cohorts was determined to be 1.98 × 10^-6^ with an odds ratio of 0.53 (Table 2). In order to exclude the possibility that the statistical interaction was driven by a direct influence of rs257174 on the risk of AR, we also tested this SNP for primary association with disease presentation. While a weak association of rs257174 with AR risk was detected in the discovery cohort (*P* = 0.01, OR = 1.32), neither the validation cohort (*P* = 0.35, OR = 0.91) nor the combined cohort produced any statistical significance (*P* = 0.38, OR = 1.07), indicating that the effect of rs257174 on incidence of AR is evident only when the variant is considered in combination with rs7071836 (marginal P-values and ORs are provided in Additional file 6: Table S3).

**Table 2:** Summary of the statistical interaction between rs7071836 and rs257174 across all cohorts

		**Discovery**		**Validation**		**Combined**	
**SNP1**	**SNP2**	**OR**^**a**^_***interaction ***_**[CI**^**b **^**95%]**	***P***_***interaction***_	**OR**^**a**^_***interaction ***_**[CI**^**b **^**95%]**	***P***_***interaction***_	**OR**^**a**^_***interaction ***_**[CI**^**b **^**95%]**	***P***_***interaction***_
rs7071836	rs257174	0.45 [0.31- 0.66]	**3.64** **×** **10**^**-5**^	0.59 [0.41-0.84]	**4.02** **×** **10**^**-3**^	0.53 [0.41- 0.69]	**1.98** **×** **10**^**-6**^

^a^Odds Ratio. ^b^Confidence Interval.

### Epistatic interaction between rs7071836 and rs257174 is associated with AR risk but not atopy

In the tropical urban environment of Singapore AR is strongly associated with the sensitization against house dust mite (HDM) allergens [35]. We therefore sought to determine whether the epistatic effect of rs257174 and rs7071836 was associated with the production of HDM-specific IgE (atopy), the key mediator of AR pathology, or rather with a downstream event influencing the manifestation of the clinical symptoms. All AR cases were atopic (HDM-IgE positive as defined by skin prick test), whereas all non-symptomatic controls were non-atopic. We therefore used the replication cohort described in [19] to assess the putative epistatic interaction by comparing the atopic AR group (AR+) comprising 676 individuals with 1647 individuals that were also atopic but did not show any AR symptoms (AR-). Epistasis of rs257174 and rs7071836 was still evident when comparing atopic AR + cases with atopic AR- cases (*P* = 9.6 × 10^-3^), but this effect was lost when comparing atopic AR- individuals with a healthy non-atopic control group comprising 511 individuals (*P* = 0.34) (Table 3). These data indicated that epistasis of rs7071836 and rs257174 affects the manifestation of clinical symptoms but does not associate with the production of HDM specific IgE.

**Table 3:** Atopy contribution to the statistical interaction between rs7071836 and rs257174 in the validation cohort

**SNP1**	**SNP2**	**AR cases**	**Samples with atopy but no AR**	**OR**^**a **^**[CI**^**b **^**95%]**	**P-value**
rs7071836	rs257174	676	1647	0.69 [0.52-0.91]	**9.60** **×** **10**^**-3**^
**SNP1**	**SNP2**	**Samples with atopy but no AR**	**Non atopic samples**	**OR**^**a **^**[CI**^**b **^**95%]**	**P-value**
rs7071836	rs257174	1647	511	0.87 [0.66-1.15]	0.34

^a^Odds Ratio. ^b^Confidence Interval.

### SNP rs257174 influences FAM134B expression levels in monocytes

**Figure 3:**
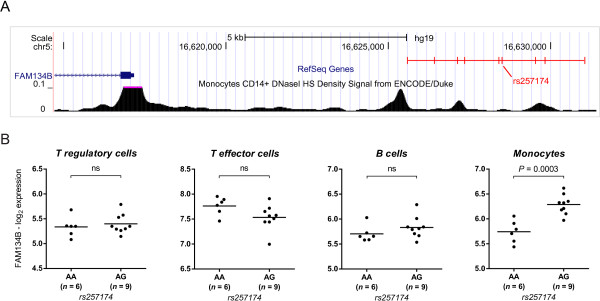
**Variations in monocyte expression of FAM134B are associated with SNP rs257174. (A)** The position of the SNP-cluster which is in perfect linkage disequilibrium (r^2^ = 1) with rs257174 in Asian individuals (CHB/JPT) is shown here with respect to the transcripts of the gene FAM134B. This SNP-cluster contains 9 SNPs located ~9 kbp upstream of the transcriptional start site. The position of the tag SNP rs257174 is indicated. Several SNPs of this cluster are located in a region of open chromatin (identified by DNase I Hypersensitivity site analysis of CD14+ monocytes in the Encode Project), suggesting a regulatory role on gene expression. **(B)** Impact of rs257174 on FAM134B gene expression in Treg, CD4+ T effector cells, B cells and monocytes (same samples as in Figure 2B). FAM134B mRNA content is plotted with reference to rs257174 genotype. Statistical analysis revealed that the polymorphism is associated with FAM134B gene expression in monocytes only (*P =* 0.0003). Gene expression was measured using the Affymetrix Human Exon 1.0ST Array and data were normalized using the Robust Multichip Average (RMA) at the gene level. P-values for the association were evaluated using one-way ANOVA on log_2_-transformed data (*n* = 15).

While statistical analysis of the GWAS data revealed an epistatic interaction of rs7071836 with rs257174, it still had to be shown that the SNP was actually associated with function. Polymorphism rs257174 is located ~12 kbp upstream of the FAM134B gene. Like its epistatic partner rs7071836, it belongs to a cluster of perfectly-linked SNPs (Figure 3A). To establish a quantitative effect on FAM134B gene expression we used mRNA samples from the same cell sources as in our rs7071836 analyses (Figure 2B). The analysis revealed that the rs257174-cluster indeed represents an ‘expression quantitative trait locus’ (eQTL). As in the case of the rs7071836-cluster the allelic effect was cell-type specific. Surprisingly, however, it was most evident in monocytes while the expression of FAM134B in Treg cells was unaffected (Figure 3B).

In-line with our findings, the influence of rs257174 on FAM134B expression has also been detected by other investigators conducting transcriptome studies of human monocytes [36,37]. For this cell type, 3 of the SNPs in the rs257174-cluster are located in close proximity to an open chromatin region (DNase I Hypersensitivity peak), which, by ENCODE criteria, suggests a functional role in the regulation of gene expression [38] (Figure 3A).

### CD39 expression is negatively correlated with FAM134B expression in whole blood

**Figure 4:**
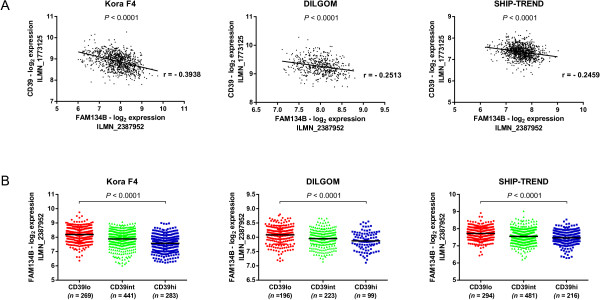
**CD39 expression negatively correlates with FAM134B expression in whole blood. (A)** Correlation between CD39 and FAM134B expression in whole blood in three Caucasian cohorts. The most significant FAM134B Illumina probe is plotted here with respect to the unique CD39 Illumina probe. A consistent negative correlation between the two genes was apparent in all cohorts (*P* < 0.001). **(B)** FAM134B expression is affected by CD39 levels in whole blood. Samples were classified according to their CD39 expression levels (CD39lo, CD39int, CD39hi) using unsupervised clustering (see Additional file 7: Figure S4) and FAM134B expression across the three groups was compared using Kruskal-Wallis test followed by Dunn’s multiple comparison tests. CD39hi individuals were characterized by a significantly lower FAM134B expression than CD39lo individuals (*P*_*krusal-wallis*_ < 0.0001, *P*_*CD39lo vs CD39hi*_ < 0.0001).

The epistatic interaction between rs7071836 and rs257174 suggested an inverse relation between the expression of CD39 and FAM134B. In order to confirm the relevance of this finding the expression levels of CD39 and FAM134B were compared in three Caucasian cohorts of healthy individuals (Kora F4 [20], DILGOM [21], SHIP-TREND [22]) for which whole blood gene expression measurements were published. In all three cohorts CD39 expression values negatively correlated with FAM134B expression values (*P*_spearman_ < 0.001, r_Kora F4_ = - 0.3938, r_DILGOM_ = - 0.2513, r_SHIP-TREND_ = -0.2459) (Figure 4A). Similarly, FAM134B was differentially expressed across samples clustered according to their CD39 expression (CD39lo, CD39int, CD39hi, Additional file 7: Figure S4). Thus, a high expression of CD39 was inversely correlated to the expression of FAM134B (Figure 4B).

### Extracellular ATP enhances FAM134B expression

**Figure 5:**
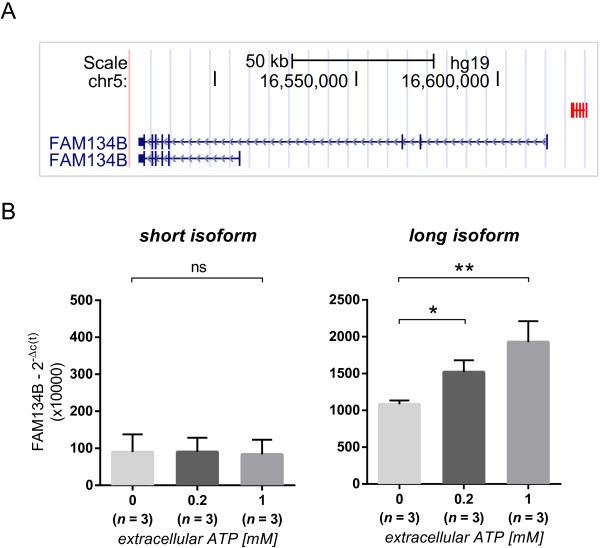
**Extracellular ATP enhances monocyte expression of FAM134B. (A)** Location of the rs257174 SNP-cluster with reference to the two common splice variants of FAM134B. The FAM134B transcript exists in two isoforms, a long isoform (~9 kbp down-stream of the rs257174 SNP-cluster), and a less abundant short isoform (more than 100 kbp distal from the rs257174 SNP-cluster). **(B)** CD14+ monocytes were isolated from 3 separate individuals and exposed to variable doses of ATP (0, 0.2 and 1 mM). After 2 h incubation, expression of FAM134B was measured by RT-PCR and normalized by the 2^-∆c(t)^ method using HPRT as the housekeeping reference gene. FAM134B expression in monocytes was significantly and dose-dependently up-regulated in response to extracellular ATP treatment. Differences between the 3 treatments were estimated using repeated measures ANOVA (*P* = 0.0089) with Tukey’s post-hoc comparison test on ∆c(t) values. Error bars represent standard deviation across biological replicates.

Since variations in CD39 Treg expression affect the concentration of ATP in the extracellular space [11], we hypothesized that this molecule could represent the functional link for the epistasis of CD39 and FAM134B. This would imply however, that FAM134B expression would be controlled by the amount of extracellular ATP. In order to test this hypothesis, we isolated CD14+ blood monocytes and incubated these cells for 2 h in the presence or absence of ATP before assessing mRNA levels of FAM134B (Figure 5A). The experiment confirmed that extracellular ATP indeed enhances the expression of FAM134B (Figure 5B). In monocytes exposed to either 0.2 mM or 1 mM ATP we observed a significant increase in FAM134B mRNA (*P =* 0.0089). Specificity of the ATP-mediated effect was confirmed by a partial block of the FAM134B expression by the addition of the ATP-antagonist A-438079 (Additional file 8: Figure S5). The stimulatory effect of ATP on FAM134B expression was dose-dependent (*P* = 0.0035, post test for assessment of linear trends), and observed only for the long isoform of FAM134B, which is encoded ~9 kbp downstream of the rs257174 LD block. Expression levels of a less abundant shorter isoform, which is encoded more than 100 kbp distal from the rs257174 LD cluster, were not affected by ATP exposure. Thus, in human monocytes the expression of FAM134B is modulated both by a genetic *cis* polymorphism and by extracellular ATP levels.

## Discussion

In this study we provide the first evidence that intercellular epistatic interactions can influence risk of complex human diseases. In this case, rs7071836, which modulated CD39 expression by human Treg, interacted with rs257174, which altered FAM134B gene expression in blood monocytes to affect the risk of AR. Both are functionally connected via extracellular ATP, a damage-associated molecule involved in eliciting a range of host inflammatory responses.

In a cohort of Singapore Chinese volunteers this novel epistatic interaction is associated to risk of allergic rhinitis. With an estimated disease prevalence of 13% [39] and an OR of 0.45, our study exhibited only 5% power to detect our CD39/FAM134B interaction in the discovery cohort at a significance threshold α = 5 × 10^-8^. We therefore elected for a replication approach to improve the power of the study. By this approach, our power for detection was increased to 89% (α = 0.0025 after Bonferroni correction for 20 tests) with a combined P-value of 1.98 × 10^-6^. While the epistasis was anchored on rs7071836, an eQTL known to regulate the surface expression of CD39, the epistasis-partner revealed by the statistical analysis also turned out to be functional. Polymorphism rs257174 was part of a SNP-cluster associated with the expression of the *cis*-Golgi protein FAM134B. Moreover, while the allelic combination of the epistasis suggested that the expression of CD39 and of FAM134B were inversely linked, this correlation could actually be validated from the expression data of a full blood transcriptome analysis.

CD39 is an ecto-ATPase that depletes extracellular ATP from the local microenvironment. Since extracellular ATP is a key indicator of host tissue damage, the efficacy of ATP removal by CD39 has been associated with various inflammatory conditions including autoimmune diseases, viral infections and cancer progression. The CD39 promoter SNP rs7071836 responsible for the effect is part of a large cluster of perfectly linked SNPs containing polymorphisms already implicated in Crohn’s disease [15] and in progression of AIDS [17]. Our study indicates that in conjunction with a cluster controlling FAM134B it is also an important component of AR risk. While the biological function of CD39 is well-established, the exact role played by the epistatic partner remains enigmatic. As a newly identified *cis*-Golgi protein, it has only been shown to be expressed on few cell types mostly associated with the neural system [40-42]. While the protein was detected in autonomic and sensory ganglia, deleterious mutations in this gene have been shown to cause hereditary sensory and autonomic neuropathy type II (HSAN II), a severe genetic disease characterized by a dysfunction of the autonomic system and impaired nociception [40]. FAM134B knock-down in a mouse N2a neuroblastoma cell line resulted in a smaller *cis*-Golgi compartment and impaired cell proliferation, and FAM134B knock-down in cultured dorsal root ganglion mouse neurons resulted in apoptosis of nociceptive neurons [40]. FAM134B may therefore be involved in mediating multiple cellular pathways that affect the maturation and export of protein precursors and cell surface receptors.

Although FAM134B has been primarily associated with the nervous system, the expression of this gene is in fact far more widespread. Overexpression of FAM134B has been reported before in human esophageal squamous cell carcinoma [43] and this study provides evidence for a constitutive expression in multiple different populations of human leukocytes. The FAM134B molecule thus seems likely to play a significant role in host immune protection and inflammatory responses. Further work will now be required to fully dissect the role played by FAM134B in the numerous different leukocyte subsets that comprise the human immune system. Considering that FAM134B is located in the *cis*-Golgi compartment, this protein could potentially be involved in vesicle trafficking and may influence cytokine secretion by monocytes in response to external stimuli including ATP.

## Conclusions

**Figure 6:**
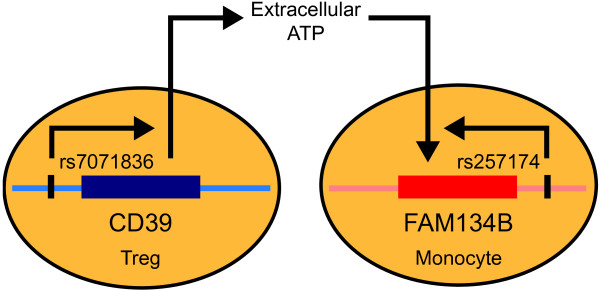
**Schematic representation of the epistatic interaction between SNPs rs7071836 and rs257174.** While promoter SNP rs7071836 regulates CD39 expression in Treg, the allelic state of rs257174 determines basal expression of FAM134B in monocytes. CD39 depletes extracellular ATP by hydrolyzing this damage-associated molecular pattern but the extent of ATP depletion depends directly on the allelic state of rs7071836. In turn, monocyte basal expression of FAM134B is regulated by rs257174 and is modulated by the concentration of ATP in the extracellular space. Host cell damage-associated ATP is thus the putative functional link that supports epistatic interaction of rs7071836 and rs257174 in determining AR risk.

Epistasis has been recognized as a natural phenomenon that commonly occurs between SNPs that affect components of the same biological pathway [44-46]. Here we propose a novel mechanism of epistasis based on the interaction of two ‘unrelated’ molecules that are regulated by polymorphisms in different cell types. Hence, epistasis can also arise from functional links that facilitate cross-talk between disparate biological pathways. In the current report, the putative mediator of this inter-lineage epistasis is ATP. While it modulates monocyte expression of FAM134B, it is also depleted from the environment by Treg via the ectonucleotidase CD39, whose expression is influenced by rs7071836 (Figure 6). The amount of FAM134B in monocytes is thus modulated both by a monocyte-specific *cis* polymorphism that determines basal expression levels and by a *trans*-polymorphism that affects CD39 expression in Treg. To our knowledge, this is the first report that intercellular genetic epistasis can play a role in susceptibility to a complex human disease.

## Abbreviations

Treg: regulatory T cells; AR: Allergic Rhinitis; SNP: Single Nucleotide Polymorphism; LD: Linkage Disequilibrium; ATP: Adenosine Triphosphate; HDM: House Dust Mite; HPRT: Hypoxanthine Guanine Phosphoribosyltransferase; IgE: Immunoglobulin E; PBMC: Peripheral Blood Mononuclear Cell.

## Competing interests

The authors declare that they have no competing interests.

## Authors’ contributions

Author contributions: RM, KJP, AKA, MS, MP and OR designed the research; KJP, TYP, MS, LZ, KP, TSL and AL performed the biological experiments; FTC, DYW and AKA collected the samples and the epidemiological data and supervised the genotyping; RM analyzed the data; RM, KJP, AKA, TYP, MP and OR wrote the paper; all the authors read and critically reviewed the manuscript.

## Pre-publication history

The pre-publication history for this paper can be accessed here:

http://www.biomedcentral.com/1471-2350/15/73/prepub

## Supplementary Material

Additional file 1: Figure S1. Gating strategy for Treg, CD4+ T effector cells, B cells and monocytes. 1–2 × 10^6^ PBMCs from each donor were stained using the LIVE/DEAD Fixable Blue Dead kit followed by surface staining with anti-CD4, CD25, CD39, CD45RA and CCR6 mAb, FoxP3 and CTLA-4 were stained intracellularly as described in Materials and methods. Monocytes and B cells constitutively expressed CD39 at steady-state. B cells were gated based on CD4-, CD39+, and CD45RA + cells whereas effector CD4+ T cells (Teff) were CD4+ and FoxP3-. CD39 was not expressed on CD45RA + or CD45RA- Teff cells. (A) rs7071836 GG genotype resulted in a higher CD39 expression on a specific subset of Treg cells, CD45RA- FoxP3+ Treg (CD45RO+). (B) In contrast, rs7071836 AA genotype was associated with a CD39 low phenotype on the same population of Treg cells. Click here for file

Additional file 2: Figure S2. Variation in CD39 cell surface expression in Caucasian and Chinese populations. CD39 expression is stable in human B cells (see Figure 1) but variable in Treg. Surface expression of CD39 in Treg was therefore normalized to the expression levels observed in donor-matched B cells in order to reduce possible batch effects. CD39 cell surface expression on the two subsets was determined by FACS analysis and individuals were clustered into two groups: CD39lo and CD39hi. Donor classification was confirmed using the unsupervised clustering *k*-means method on log_2_-transformed ratios, setting the number of clusters to two. While no major differences in clustering were observed between ethnicities, the relative frequency of CD39lo individuals was higher in the Chinese cohort. Click here for file

Additional file 3: Figure S3. FACS Sorting strategy for Teff, Treg, B cells, and monocytes. (A) Gating strategy for the pre-sort sample. PBMCs were incubated with CD49d, CD127, CD4, CD25, CD19, and CCR6 mAbs in MACS buffer for 15 min. After staining, the samples were washed with MACS buffer and re-suspended at 40–50 × 10^6^ cells per ml in MACS buffer and applied to 70 uM Pre-separation filters (Miltenyi Biotec). The filtered samples were applied to a FACSAriaII cell sorter. Teff, Treg, B cells were collected in 5 ml Falcon polystyrene tube containing 1 ml of FACS sorting media (RPMI1640 supplemented with 10% fetal bovine serum and 10 μg/ml gentamicin). For Treg cells, these cells were collected in 1.5 ml Eppendorf tube containing 1 ml of FACS sorting media. (B) Post-sort analysis of Treg, Teff, B cell, and monocyte. Post sort analysis was performed to determine the purity of Treg, Teff, B cells, and monocytes. Click here for file

Additional file 4: Table S1. CD39 epistatic interactions identified in the discovery cohort (*P* < 0.0001). Click here for file

Additional file 5: Table S2. CD39 epistatic interactions tested in the replication cohort. Click here for file

Additional file 6: Table S3. Marginal and interaction P-values and ORs for the statistical interaction between rs7071836 and rs257174 across all cohorts. Click here for file

Additional file 7: Figure S4. CD39 expression clustering in whole blood to simulate the effect of polymorphism rs7071836. Gene expression processed data were downloaded from the respective repositories and used as input for the clustering algorithm *k*-means. The number of clusters was set to three. Significance was assessed using Kruskal-Wallis test followed by Dunn’s multiple comparison tests. Each cohort was analyzed independently. In all cohorts the three CD39 expression clusters (CD39lo, CD39int, CD39hi) were strongly separated. Click here for file

Additional file 8: Figure S5. Inhibition of the ATP-mediated induction of FAM134B by a purinergic receptor antagonist. Monocytes of two donors were incubated for 2 h with 1 mM ATP in the absence or presence of the ATP antagonist 10 μM A-438079. After the incubation the mRNA levels of FAM134B were determined by RT-PCR. The analysis revealed a 50% and 36% reduction in reference to the basal expression in the absence of ATP (red dashed line). Click here for file
