# Supplementary figures and images for: Genetic analysis of an allergic rhinitis cohort reveals an intercellular epistasis between FAM134B and CD39
Source: BMC Med Genet. 2014 Jun 27;15:73. doi: 10.1186/1471-2350-15-73 (PMC4094447; doi:10.1186/1471-2350-15-73)

A

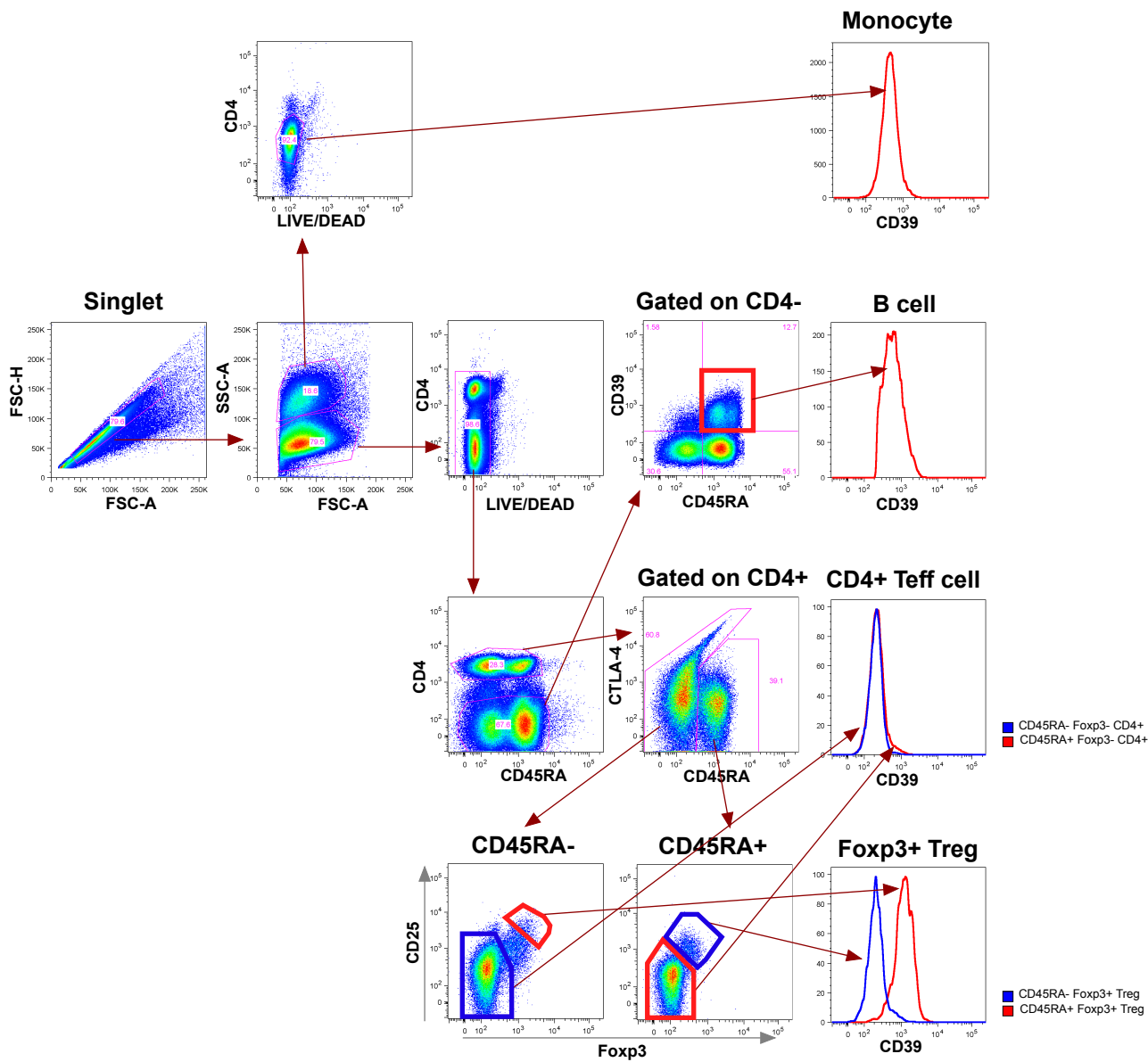

**rs7071836:GG**  
**(“CD39hi”)**

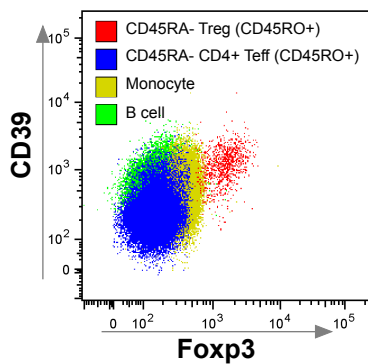

# B

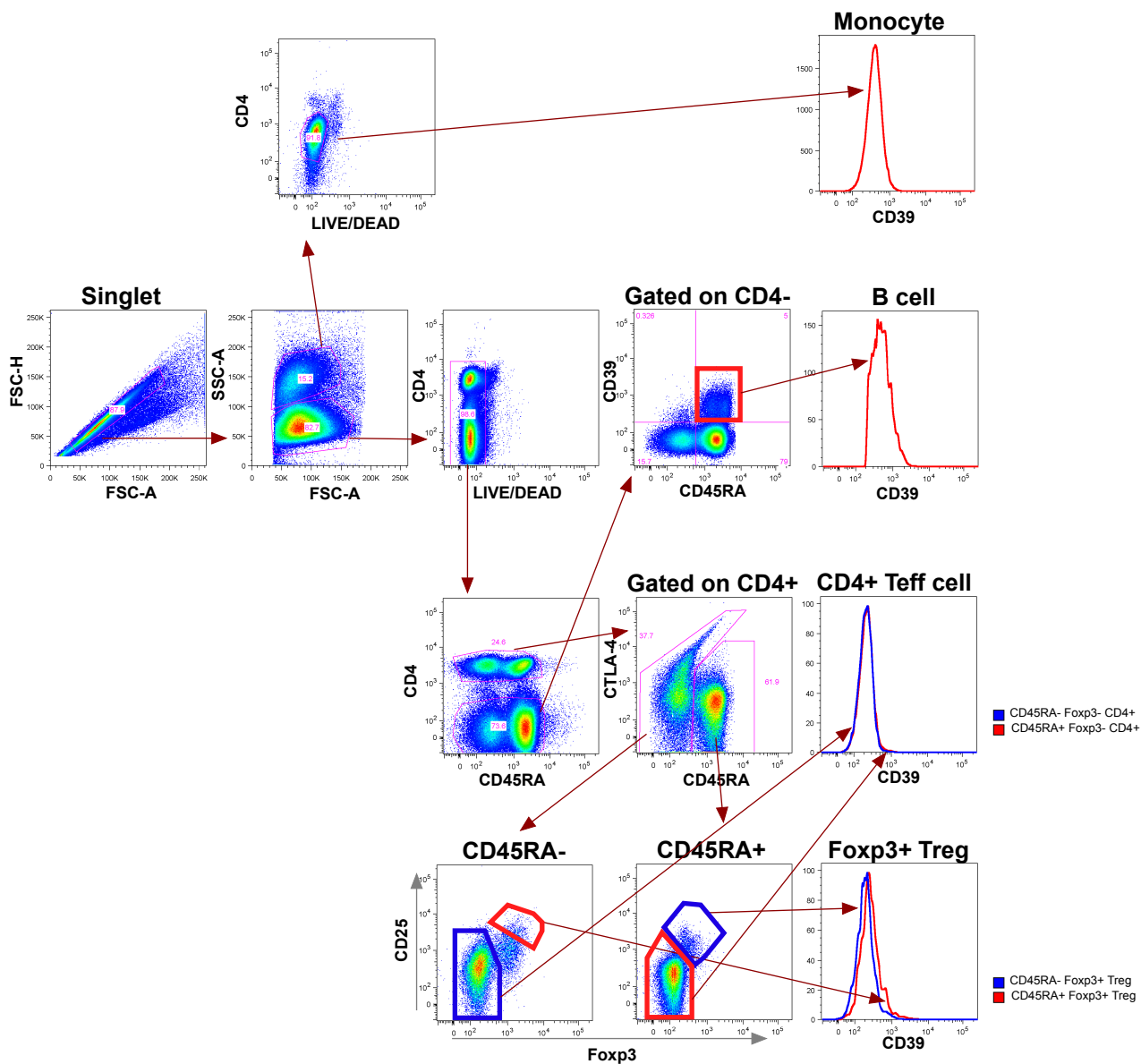

**rs7071836:AA**  
**("CD39lo")**

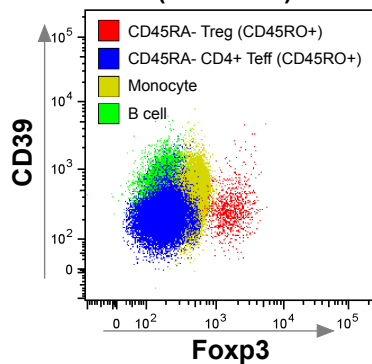

Supplement: Additional file 1: Figure S1. — Gating strategy for Treg, CD4+ T effector cells, B cells and monocytes. 1–2 × 10^6^ PBMCs from each donor were stained using the LIVE/DEAD Fixable Blue Dead kit followed by surface staining with anti-CD4, CD25, CD39, CD45RA and CCR6 mAb, FoxP3 and CTLA-4 were stained intracellularly as described in Materials and methods. Monocytes and B cells constitutively expressed CD39 at steady-state. B cells were gated based on CD4-, CD39+, and CD45RA + cells whereas effector CD4+ T cells (Teff) were CD4+ and FoxP3-. CD39 was not expressed on CD45RA + or CD45RA- Teff cells. (A) rs7071836 GG genotype resulted in a higher CD39 expression on a specific subset of Treg cells, CD45RA- FoxP3+ Treg (CD45RO+). (B) In contrast, rs7071836 AA genotype was associated with a CD39 low phenotype on the same population of Treg cells. [file 1471-2350-15-73-S1.pdf]

***Caucasian (n = 22)***

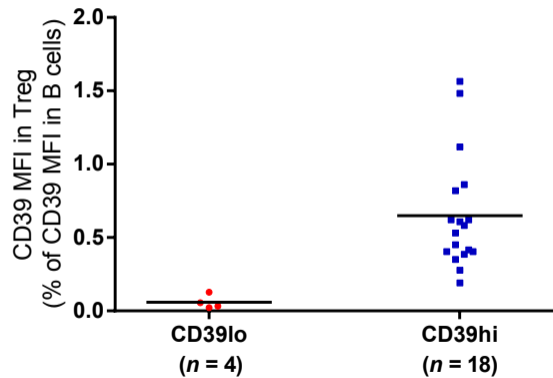

***Chinese (n = 41)***

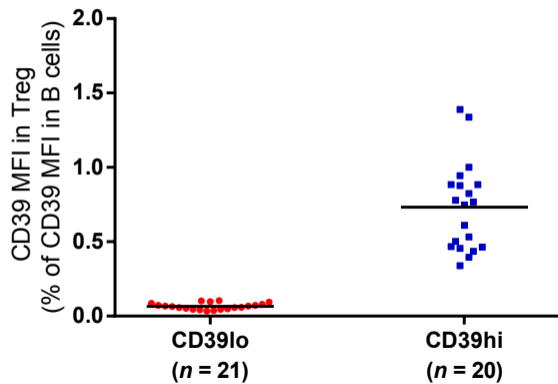

Supplement: Additional file 2: Figure S2. — Variation in CD39 cell surface expression in Caucasian and Chinese populations. CD39 expression is stable in human B cells (see Figure 1) but variable in Treg. Surface expression of CD39 in Treg was therefore normalized to the expression levels observed in donor-matched B cells in order to reduce possible batch effects. CD39 cell surface expression on the two subsets was determined by FACS analysis and individuals were clustered into two groups: CD39lo and CD39hi. Donor classification was confirmed using the unsupervised clustering k-means method on log2-transformed ratios, setting the number of clusters to two. While no major differences in clustering were observed between ethnicities, the relative frequency of CD39lo individuals was higher in the Chinese cohort. [file 1471-2350-15-73-S2.pdf]

A

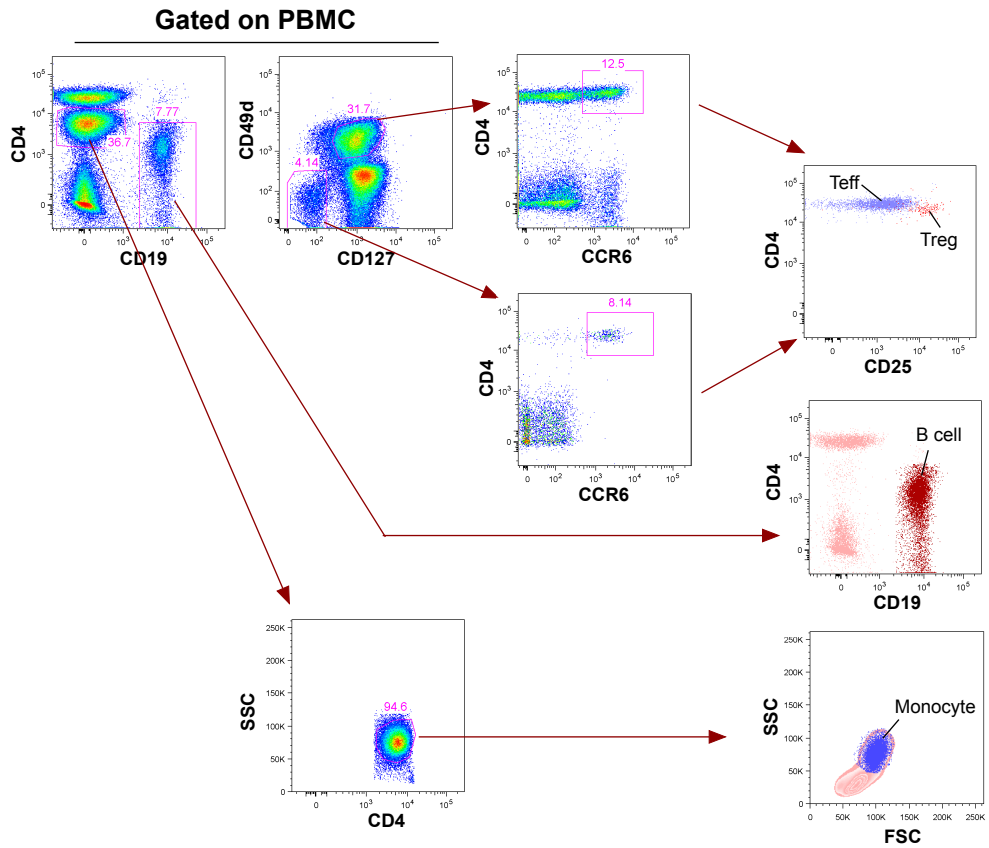

B

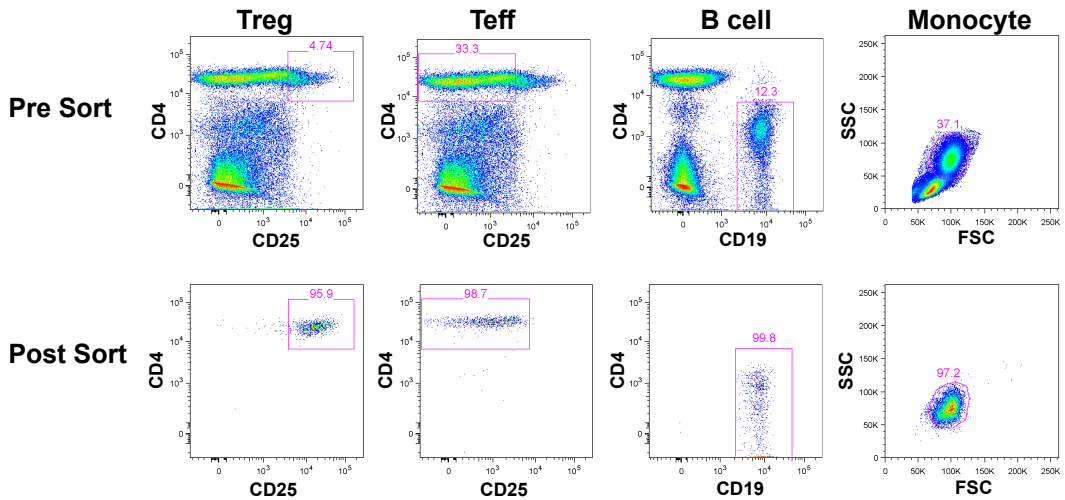

Supplement: Additional file 3: Figure S3. — FACS Sorting strategy for Teff, Treg, B cells, and monocytes. (A) Gating strategy for the pre-sort sample. PBMCs were incubated with CD49d, CD127, CD4, CD25, CD19, and CCR6 mAbs in MACS buffer for 15 min. After staining, the samples were washed with MACS buffer and re-suspended at 40–50 × 106 cells per ml in MACS buffer and applied to 70 uM Pre-separation filters (Miltenyi Biotec). The filtered samples were applied to a FACSAriaII cell sorter. Teff, Treg, B cells were collected in 5 ml Falcon polystyrene tube containing 1 ml of FACS sorting media (RPMI1640 supplemented with 10% fetal bovine serum and 10 μg/ml gentamicin). For Treg cells, these cells were collected in 1.5 ml Eppendorf tube containing 1 ml of FACS sorting media. (B) Post-sort analysis of Treg, Teff, B cell, and monocyte. Post sort analysis was performed to determine the purity of Treg, Teff, B cells, and monocytes. [file 1471-2350-15-73-S3.pdf]

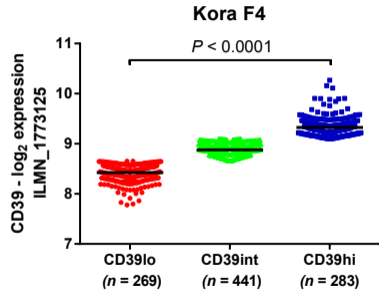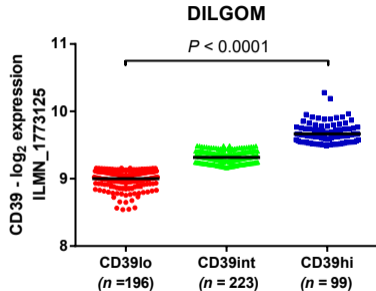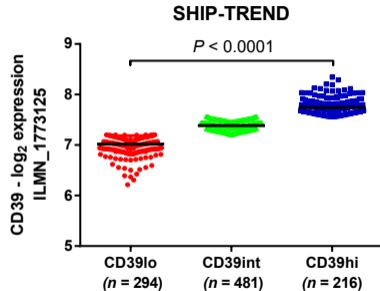

Supplement: Additional file 7: Figure S4. — CD39 expression clustering in whole blood to simulate the effect of polymorphism rs7071836. Gene expression processed data were downloaded from the respective repositories and used as input for the clustering algorithm k-means. The number of clusters was set to three. Significance was assessed using Kruskal-Wallis test followed by Dunn’s multiple comparison tests. Each cohort was analyzed independently. In all cohorts the three CD39 expression clusters (CD39lo, CD39int, CD39hi) were strongly separated. [file 1471-2350-15-73-S7.pdf]

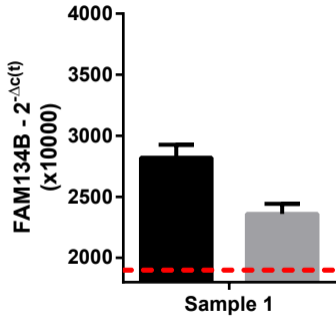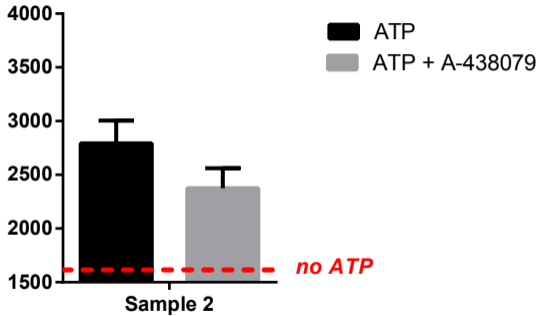

Supplement: Additional file 8: Figure S5. — Inhibition of the ATP-mediated induction of FAM134B by a purinergic receptor antagonist. Monocytes of two donors were incubated for 2 h with 1 mM ATP in the absence or presence of the ATP antagonist 10 μM A-438079. After the incubation the mRNA levels of FAM134B were determined by RT-PCR. The analysis revealed a 50% and 36% reduction in reference to the basal expression in the absence of ATP (red dashed line). [file 1471-2350-15-73-S8.pdf]
