# Supplementary material for: Genetic analysis of an allergic rhinitis cohort reveals an intercellular epistasis between FAM134B and CD39
Source: BMC Med Genet. 2014 Jun 27;15:73. doi: 10.1186/1471-2350-15-73 (PMC4094447; doi:10.1186/1471-2350-15-73)
Supplement: Additional file 6: Table S3. — Marginal and interaction P-values and ORs for the statistical interaction between rs7071836 and rs257174 across all cohorts. [file 1471-2350-15-73-S6.doc]

|  | **rs7071836** | | **rs257174** | | **Interaction** | |
| --- | --- | --- | --- | --- | --- | --- |
|  | **OR^a^**  **[CI^b^ 95%]** | ***P*** | **OR^a^**  **[CI^b^ 95%]** | ***P*** | **OR^a^**  **[CI^b^ 95%]** | ***P*** |
| **Discovery** | 1.47  [1.11-1.93] | 6.42 x 10^-3^ | 2.01  [1.48-2.71] | 6.18 x 10^-6^ | 0.45  [0.31-0.66] | 3.64 x 10^-5^ |
| **Validation** | 1.06  [0.84-1.34] | 0.62 | 1.15  [0.88-1.51] | 0.30 | 0.59  [0.41-0.84] | 4.02 x 10^-3^ |
| **Combined** | 1.21  [1.01-1.44] | 3.92 x 10^-2^ | 1.46  [1.20-1.78] | 2.05 x 10^-4^ | 0.53  [0.41-0.69] | 1.98 x 10^-6^ |

^a^Odds Ratio. ^b^Confidence Interval.
